# Optimal perioperative care in peri-hilar cholangiocarcinoma resection

**DOI:** 10.1007/s10353-018-0529-x

**Published:** 2018-05-04

**Authors:** Leonard M. Quinn, Declan F. J. Dunne, Robert P. Jones, Graeme J. Poston, Hassan Z. Malik, Stephen W. Fenwick

**Affiliations:** 1 Liverpool Hepatobiliary Centre, Aintree University Hospital, Longmoor Lane, L9 7AL Liverpool, UK; 2 Institute of translational Medicine, University of Liverpool, Ashton Street, L69 3GE Liverpool, UK

**Keywords:** Cholangiocarcinoma, Peri-operative care, Pre-operative fitness assessment, Pre-habilitation, Enhanced Recovery

## Abstract

Surgical resection remains the only proven curative treatment for peri-hilar cholangiocarcinoma. Despite recent advances in liver surgery techniques and perioperative care, resection for peri-hilar cholangiocarcinoma remains associated with significant morbidity and mortality. Considerable variation in the perioperative management of these patients exists. Optimal perioperative management has the potential to deliver improved outcomes. This article seeks to summarize the evidence underpinning best practice in the perioperative care of patients undergoing resection of peri-hilar cholangiocarcinoma. The authors also seek to identify areas where research efforts and future clinical trials should be targeted.

## Introduction

Complete surgical resection represents the only curative option in peri-hilar cholangiocarcinoma (pCCA); however, the disease is often silent in the early stages and many patients have advanced disease at presentation. The majority of patients undergoing resection do not achieve long-term disease control, but radical curative resection can achieve a 5-year survival of 11 to 44% [1]. Surgical resection represents a major undertaking, with mortality in western specialist centres ranging from 13% [2] to 15% [3], and morbidity of up to 40% [4]. Research to improve postoperative outcomes through optimized perioperative management is urgently needed to reduce this mortality and morbidity burden and minimize management variation. This article seeks to summarize the evidence underpinning best practice in the perioperative care of patients undergoing resection of peri-hilar cholangiocarcinoma.

## Cholangiocarcinoma

Cholangiocarcinoma represents a spectrum of biliary tract adenocarcinomas. The disease encompasses intra-hepatic (10%) and extra-hepatic cases, including peri-hilar disease (50%) arising at or near the confluence of the hepatic ducts, and distal disease (40%; [5]).

Patients with surgically resectable disease enjoy the most favourable prognosis, and this is a key determinate in the staging of pCCA [6]. The most commonly used staging system is the Union for International Cancer Control/American Joint Committee on Cancer (UICC/AJCC) 2010 revision of the tumour, node, metastasis (TNM) classification, separating cholangiocarcinoma into intra-hepatic, hilar and distal disease, respectively [7, p. 201].

None of the staging systems accurately predict survival. The most important staging and predictive issue is surgical resectability. The AJCC system is based on pathological outcome following resection.

Clinical preoperative staging systems for pCCA include the Bismuth–Corlette and Memorial Sloan Kettering Cancer Centre (MSKCC) systems. Bismuth–Corlette classifies patients on the extent of biliary involvement but does not incorporate vascular involvement or lobar atrophy. As such, it cannot be used for predicting resectability. MSKCC builds on Bismuth–Corlette and includes longitudinal and radial extension of the tumour to more accurately predict resectability. T staging includes local tumour involvement, portal vein involvement and hepatic lobar atrophy. This staging system has been externally validated and accurately predicts resectability, probability of metastatic disease and long-term survival in the preoperative setting [6].

Resectability is ultimately determined at the time of surgery, as these tumours often extend into the liver and major vascular structures, with accurate preoperative evaluation of these areas difficult. Therefore, surgical exploration with or without trial dissection is appropriate for potentially resectable disease based on preoperative imaging [8].

For pCCA, bile duct resection alone results in high local recurrence rates [9]. The addition of hepatic resection improves R0 resection rate [10]. R0 resection is the best management option for prolonged survival, where technically feasible [11].

Neoadjuvant therapy and liver transplantation are not considered standard of care at the current time. The Mayo Clinic considers liver transplantation in highly selected cases of early-stage local unresectable peri-hilar CCA in patients who have completed thorough staging, assessment and neoadjuvant chemoradiotherapy [12].

The published Mayo experience found a statistically significant improved survival following their transplantation protocol with 92% 1‑year, 82% 3‑year and 82% 5‑year survival. This compared to 82%, 48% and 21%, respectively, following resection only [13]. The ongoing French Phase III Transphil trial compares preoperative capecitabine and radiotherapy followed by liver transplantation with standard surgical resection (NCT 02232932).

## Biliary drainage

Jaundice is often the first presenting symptom of pCCA, and whether preoperative biliary decompression should be performed remains controversial [14].

Ribero D et al., found that preoperative cholangitis and insufficient functional residual liver volume are the major determinants of hepatic insufficiency and liver failure-related death. Given the association between biliary drainage and cholangitis, they advocate that the preoperative approach to patients with peri-hilar CCA should be optimized to minimize the risk of cholangitis [15].

A number of recent meta-analyses have focused on this issue. Moole H et al. found that patients with malignant biliary jaundice requiring surgery who underwent biliary decompression had significantly less major adverse effects those who went directly to surgery. However, this study included all malignant causes of biliary obstruction [16].

The optimal method (endoscopic versus percutaneous transhepatic) by which to perform preoperative biliary drainage is debated. The Dutch multicentre DRAINAGE trial was designed to identify a difference in the number of severe drainage-related complications between endoscopic and percutaneous transhepatic approaches in patients with pCCA selected to undergo major liver resection. The trial hypothesized that less complications would occur in the percutaneous group. This trial identified an increased morbidity risk in the percutaneous group and was subsequently prematurely terminated [17].

This issue remains debatable and patients are managed according to local unit protocols. Randomized trials are needed to identify those who will benefit most from biliary drainage, and the optimal method for biliary drainage. In the presence of jaundice in perihilar cholangiocarcinoma patients planned for surgery, the authors recommend preoperative biliary decompression of the planned future liver remnant by the endoscopic method. Metallic stents offer higher patency duration than plastic stents [18].

## Optimizing the future liver remnant

Postoperative hepatic failure (PHF) is a leading cause of death following liver surgery [19]. Optimizing the future liver remnant is key to maintaining adequate liver function postoperatively and minimizing the risk of PHF. Standard principles of liver resection should be maintained, including minimized blood loss, hypotension and the judicious use of hepatic inflow occlusion. Jaundice impairs regeneration by impedance of portal blood flow. Hepatic resection exacerbates this decrease in portal flow and hence regeneration. Although preoperative biliary drainage is controversial, as described previously, adequate drainage of the future liver remnant is deemed a necessity if it is to be a small liver remnant [20]. If the liver volume is below threshold, then regeneration ceases, as other metabolic functions assume priority [21]. A continued imbalance will lead to liver failure. These issues are of overriding importance in peri-hilar resection, where extended resection and caudate lobectomy may be indicated [22].

If the future liver remnant is deemed inadequate on preoperative assessment, then portal venous embolization (PVE) can be considered. Occlusion of portal vein branches to liver that is to be resected can induce lobar hypertrophy in the future liver remnant. This is assessed by CT volumetric analysis. Portal venous embolization can increase the remnant volume with low morbidity [23]. Thus, PVE has a potential benefit for patients with advanced biliary cancer who are to undergo extended, complex hepatectomy [24]. There are a number of non-randomised studies; there are, however, no randomized data currently available for this intervention.

A recent case–control analysis of the potential role of ALPPS (associating liver partition and portal vein ligation for staged hepatectomy) for extended hepatectomy in pCCA resection by Olthof PB et al. found inferior outcomes in ALPPS-treated patients compared to standard extended hepatectomy approaches. ALPPS is therefore not currently recommended for pCCA, and as such, portal vein embolization remains the method of choice for increasing the functional liver remnant [25].

## Staging laparoscopy

Staging laparoscopy is used in the staging of peri-hilar cholangiocarcinoma following radiological assessment to determine the presence or absence of radiologically occult metastases, thereby reducing the incidence of unnecessary laparotomy [26].

Bird et al. suggested that staging laparoscopy has a 27% all-cause yield of unresectable disease and a sensitivity of 71% for peritoneal disease [27]. However, there remains debate regarding the value of routine staging laparoscopy. Coelen et al. addressed this issue in a pooled meta-analysis of 12 studies published in 2016, suggesting that 25% of patients with potentially resectable pCCA benefit from staging laparoscopy with the highest sensitivity for peritoneal metastases [28]. They suggest that with further improvement of radiological techniques through time, it may be possible to identify patients who will benefit most from laparoscopy. Until such time, the authors recommend staging laparoscopy for exclusion of peritoneal metastases.

Coelen et al. proposed a preoperative risk score for selection into laparoscopy that demonstrated good discrimination in predicting unresectability, stratifying patients into low, intermediate and high risk (Tables 1 and 2). This scoring system has not been externally validated but may help identify those patients in whom staging laparoscopy offers most benefit [29].

**Table 1:** Pre-operative risk score to predict unresectable peri-hilar cholangiocarcinoma at staging laparoscopy

Variable	Classes	Points
*Tumour size*	<4.5 cm	0
	>4.5 cm	1
*Portal vein involvement*	None or unilateral	0
	Bilateral or main stem	1
*Suspected LN metastases*	None or N1	0
	N2	1
*Suspected extra-hepatic metastases*	No	0
	Yes	2

**Table 2:** Predicted and observed risks according to risk score

Group	Total points	Unresectability at staging Laparoscopy	
		Predicted (%)	Observed (%)
*Low risk*	0	7.2	6.4
*Intermediate risk*	1	21.3	28.2
*High risk*	2	48.5	47.6
	3	76.5	66.7
	4	91.9	100
	5	N/A	N/A

*N/A* not applicable

## Nutrition

Nutritional deficiencies in patients presenting with pCCA pose a significant risk to perioperative outcomes. The evidence base is poor due to the relative scarcity of the disease, and as a result, the management strategy must be extrapolated from other biliary tract cancers including pancreatic cancer.

Obstruction of biliary flow leads to jaundice (the most common presenting symptom) with malabsorption and maldigestion through impaired lipid emulsification [30]. Furthermore, there is the additional nutritional compromise due to biliary sepsis. The impact of biliary stenting on preoperative nutritional optimization has not been documented in cholangiocarcinoma.

The international consensus definition of cancer cachexia includes weight loss (main criterion), low muscle mass (sarcopenia) and low body mass index, and is associated with increased mortality risk. Affecting up to 80% of pancreatic cancer patients, the syndrome of cancer cachexia can also include many more pathophysiological drivers such as inflammation, altered protein metabolism, skeletal muscle loss, adipose tissue loss, anorexia, malabsorption and neurohormonal changes [31].

Sarcopenia is associated with poor prognosis in liver surgery for colorectal metastases [32]. More recently, it has been demonstrated to increase the rate of liver failure in patients undergoing major hepatectomy with extra-hepatic bile duct resection for pCCA [33]. In resection of intrahepatic cholangiocarcinoma, sarcopenia is also associated with higher postoperative mortality [34].

Causation of nutritional deficiency may vary in pCCA. Screening for causation and tailored pre- and postoperative management is essential. There have been no studies examining nutritional intervention in patients presenting with resectable pCCA and this should form a focus of urgent further research.

## Preoperative fitness assessment

The substantial physiological insult of major hepatectomy, alone or in conjunction with bile duct resection, is associated with high rates of postoperative morbidity. Identification of those patients at risk of developing significant postoperative morbidity plays a key role in the assessment of patients prior to pCCA resection.

Cardiopulmonary exercise testing (CPET) is an objective method of assessing preoperative cardiopulmonary fitness, with the aim of improving accuracy of preoperative prediction of postoperative complications and mortality. The role of CPET in liver surgery has been examined. In 104 high-risk patients undergoing hepatectomy, oxygen uptake at the anaerobic or lactate threshold (AT) was the only CPET predictor of postoperative morbidity on multivariate analysis [35]. Another study suggested that patients with higher AT have earlier hospital discharge, but that a low relative oxygen uptake at the AT did not confer a significantly higher risk of postoperative complications. This retrospective study suggested that the utilization of CPET to tailor perioperative care had minimized the impact of lower fitness, consequently suggesting that CPET-assessed poor fitness should not be deemed a barrier to surgical intervention in elective hepatectomy. CPET assessment should therefore be interpreted in the context of a wider anaesthetic review and should aid complex perioperative decision-making [36].

CPET assessment is not universally used in the preoperative assessment of pCCA. Further studies are needed to examine the role of CPET and the values of most importance when predicting the outcome of pCCA patients undergoing resection.

## Prehabilitation

Prehabilitation aims to prevent or minimize the morbidity of surgery [37]. These preoperative interventions focus on preoperative fitness, nutrition, education and preoperative psychological status [38]. In liver surgery, a CT identified that a 4-week program of exercise was capable of delivering meaningful improvements in preoperative fitness in patients prior to hepatectomy for colorectal liver metastasis [39].

There are no studies assessing prehabilitation in pCCA. The feasibility of prehabilitation in patients presenting with cholangiocarcinoma needs further investigation to establish whether it could be used to mitigate the high perioperative mortality and morbidity.

## Enhanced recovery

Enhanced recovery after surgery (ERAS) has been shown to improve perioperative outcomes and reduce cost in colorectal surgery, where it is now deemed a standard of care [40]. It is not yet standard of care in liver surgery but can be successfully implemented without compromising morbidity or mortality rates [41]. Many of the principles are derived from colorectal surgery, but distinct differences exist which may impede implementation in hepatopancreatobiliary (HPB) surgery [42]. ERAS aims to decrease variability in postoperative management, enhance quality of care and improve outcomes including length of inpatient hospital stay. ERAS protocols include greater preoperative education, preoperative oral carbohydrate loading, postoperative goal-directed therapy, early mobilization and physiotherapy.

There are several studies focused on ERAS in liver surgery. In 2013, Jones C et al. conducted a randomised controlled trial (RCT) of enhanced recovery versus standard care in open resection and found ERAS protocols safe and effective, with faster recovery and discharge in the ERAS cohort. They also reported fewer medical-related complications and improved quality of life in the ERAS-treated group [43]. Savikko J et al. safely implemented a liver ERAS protocol with discharge within 4 days and without any significant increase in adverse events in a primarily open-resected cohort published in 2015 [44]. The largest series of ERAS in liver surgery included 303 patients undergoing colorectal liver metastasis resection. This study suggested that ERAS can be universally applied, and that the benefits accrue with time [45]. It was suggested that ERAS should be considered standard of care in hepatectomy for CRLM.

Overall evidence from systematic reviews is limited, retrospective in nature and largely focused on open resections. However, they all share similar conclusions that ERAS is a safe and effective program in liver surgery. Ahmed EA et al. found that fast-track enhanced recovery programs were safe and feasible, and recommend further work on multimodal analgesia and overall adherence to the programs [46]. The meta-analysis by Zhao Y et al. included 7 RCTs and further divided the analysis into laparoscopic and open subgroups to explore the effectiveness of ERAS in these different surgical approaches. Again, ERAS programs can enhance short-term recovery after liver resection in both approaches and are safe and worthwhile. Protocol-specific ERAS guidelines for liver resection are recommended [47].

Peri-hilar cholangiocarcinoma is often excluded from series describing ERAS in liver surgery. No study has specifically addressed ERAS in the context of pCCA resection. A recent publication by Yip et al. included 27 patients with pCCA in a series of 223 patients undergoing hepatectomy [48]. Whilst this demonstrates feasibility, tailoring of ERAS programs for pCCA is urgently needed.

There are concerns specific to cholangiocarcinoma, primarily anastomotic leak and collections secondary to the formation of biliary-enteric anastomoses. As such, this procedure is associated with greater morbidity than standard liver resection. Future studies in pCCA should focus on these issues.

In view of the complications associated with peri-hilar resection, we routinely follow-up all postoperative patients within 2 weeks of resection. In addition, all patients have access to a hepatobiliary specialist nurse-led telephone clinic.

## Chemotherapy

Most patients undergoing resection for pCCA will develop recurrence. Predictors of recurrence include positive margin status, vascular invasion and lymph node metastasis [7, p. 219].

The European Society of Medical Oncology and the National Comprehensive Cancer Network suggest chemotherapy for both margin-negative and margin-positive resected patients [49]. On the basis of improved survival in periampullary cancer in the ESPAC-3 trial, gemcitabine or fluorouracil have been considered acceptable adjuvant chemotherapy for pCCA [50].

The recently reported UK BilCap Phase III trial found much extended median survival with capecitabine. In 430 patients, capecitabine was associated with a 25% lower risk of death compared to observation alone [51]. Per protocol analysis demonstrated a statistically significant survival benefit. This included large numbers of R1 resections but is deemed clinically highly relevant. This would suggest adjuvant capecitabine to be considered the new standard of care in biliary tract cancer [52].

Preoperative chemotherapy or radiotherapy is not considered routine treatment, in part due to the complications of jaundice and malnutrition. However, in selected patients there may be benefit. A small study identified complete pathological response and margin-negative resection in 3 out of 9 patients with extra-hepatic CCA [53]. Further work identified a survival benefit despite the neo-adjuvant cohort having more advanced disease [54]. These promising early findings warrant further investigation with appropriately powered clinical trials.

No prospective clinical trials have identified a benefit with adjuvant radiotherapy.

## Conclusions

Despite improvements in overall survival and advances in liver surgery, morbidity and mortality rates remain high in patients undergoing resection for peri-hilar cholangiocarcinoma.

The incidence of nutritional compromise in pCCA is unknown but likely to be high; nutritional optimization is likely to confer a benefit on postoperative outcomes. There are no primary studies assessing prehabilitation in pCCA. The feasibility of prehabilitation in patients presenting with pCCA needs investigation.

In the presence of jaundice, biliary drainage to optimize the future liver remnant and prevent cholangitis is essential. Where the functional liver remnant (FLR) will be inadequate, PVE remains the method of choice to increase remnant volume.

Staging laparoscopy is highly sensitive for the detection of radiologically occult metastases and should therefore be considered standard of care.

CPET assessment is not universally used in the preoperative cardiovascular fitness assessment of pCCA. The role of CPET in predicting outcome needs further assessment.

ERAS is not yet the standard of care in liver surgery but can be successfully and safely implemented without compromising morbidity or mortality rates. An assessment of the ERAS specific to pCCA resection focusing on the appropriate components is warranted.

The majority of patients undergoing resection for pCCA will develop recurrence. In light of the BilCap trial, the authors recommend capecitabine as the new standard of care for adjuvant chemotherapy.

There remains room for significant improvement in the perioperative management of pCCA. Optimized evidence-based perioperative management strategies represent a target to improve outcome in these patients.
